# Impacts of the Pandemic on Social Determinants of Health in an Academic Emergency Department

**DOI:** 10.5811/westjem.2022.8.56145

**Published:** 2022-11-01

**Authors:** Shannon Findlay, Uche Okoro, Sangil Lee, Karisa Harland, Marisa Evers, Elizabeth Gaffney, Mary McCormick, Chris Buresh

**Affiliations:** * The University of Iowa Roy J. and Lucille A. Carver College of Medicine, Department of Emergency Medicine, Iowa City, Iowa; † University of Michigan, Department of Emergency Medicine, Ann Arbor, Michigan; ‡ University of Washington, Department of Emergency Medicine, Seattle, Washington

## Abstract

**Introduction:**

The coronavirus 2019 (COVID-19) pandemic caused significant disruptions in daily life. Given the role that social determinants of health play in the overall well-being of individuals and populations, we wanted to determine the effects of the COVID-19 pandemic on our patient population in the emergency department (ED).

**Methods:**

We adapted the Centers for Medicare and Medicaid Services social risk assessment to assess changes to participants’ social situations throughout the COVID-19 pandemic from January 2020–February 2021. The survey was administered within the ED to individuals selected by a convenience sample of patients who were stable enough to complete the form.

**Results:**

We received 200 (66%) responses from the 305 patients approached. Worsened food access was reported by 8.5% (17) of respondents, while 13.6% (27) reported worsened food concern since the onset of the COVID-19 pandemic. The odds of worsened food access were higher among non-Whites (adjusted odds ratio [aOR] 19.17, 95% confidence interval [CI] 3.33–110.53) and females (aOR 9.77, CI 1.51–63.44). Non-Whites had greater odds of worsened food concern (aOR 15.31, CI 3.94–59.54). Worsened financial difficulty was reported by 24% (48) of respondents. The odds of worsened financial difficulty were higher among females (aOR 2.87, 95% CI 1.08–7.65) and non-Whites (aOR 10.53, CI 2.75–40.35).

**Conclusion:**

The COVID-19 pandemic has worsened many of the social determinants of health found within communities. Moreover, vulnerable communities were found to be disproportionately affected as compared to their counterparts. Understanding the challenges faced by our patient populations can serve as a guide on how to assist them more comprehensively.

## INTRODUCTION

### Background

Coronavirus 2019 (COVID-19) is an infectious respiratory disease caused by the severe acute respiratory syndrome coronavirus 2. The first COVID-19 case in the United States, according to the US Centers for Disease Control and Prevention (CDC), was reported on January 21, 2020. On March 11, 2020, the World Health Organization (WHO) declared COVID-19 to be a global pandemic. Beyond the health effects caused by the disease, COVID-19 also precipitated economic disruptions throughout the country. With this came unprecedented unemployment rates, loss of insurance, and severe economic hardships, especially among low-income populations.^1^ People diagnosed with COVID-19 have faced employment challenges and large medical bills.^2^ These sudden economic shocks can increase morbidity and mortality, especially within the realm of mental health. It has been noted that higher rates of unemployment are correlated with increased rates of suicide.^3^

The COVID-19 pandemic has further highlighted the relationship of our traditional health views with the importance of understanding the impact of social determinants of health (SDoH) on overall well-being. Moreover, the pandemic has illustrated the role of SDoH at both an individual and population health level. The SDoH, as defined by WHO, are “conditions in which people are born, grow, live, work, and age,” which are “shaped by the distribution of money, power, resources at global, national, and local levels.”^4^ The CDC further categorizes SDoH into five domains: economic stability; education access and quality; healthcare access and quality; neighborhood and built environment; and social and community context. Although separately categorized, these five domains impact one another rather than acting as isolated entities.

### Importance

With the role that SDoH play in the overall well-being of individuals and populations, the information gathered from participants’ responses to our survey helped determine the effects of the COVID-19 pandemic on their overall health. The effects of the pandemic on SDoH may also be viewed through the lens of social vulnerability. Social vulnerability refers to the potential negative effects on communities caused by external stressors on human health. Such stressors include natural or human-caused disasters, or disease outbreaks. Vulnerable and at-risk populations have been shown to be most likely to seek care through the emergency department (ED).^5^,^6^

### Objective of This Investigation

With this understanding of SDoH and social vulnerability, we conducted a survey within the ED to assess how the pandemic has impacted our patient population and its effect on social risk.

## METHODS

### Study Design and Setting

This was a cross-sectional survey evaluating changes to patients’ social situations throughout the COVID-19 pandemic. In this study we adhered to the Strengthening the Reporting of Observational studies in Epidemiology (STROBE) initiative reporting guidelines. The institutional review board approved the study under a waiver of informed consent.

The study setting included adult patients and the guardians of pediatric patients seen in an ED at a 60,000-visit, academic. Level I trauma center between October 2020–February 2021. This timeframe was impacted by the COVID-19 pandemic. The community of Iowa City in Johnson County, Iowa, has a population of approximately 74,000, consisting primarily of 78% White, 8% Black, 7% Asian, and 6% Hispanic.^7^ From October 2020–March 2021, our community’s average unemployment rate was 3.7%, vastly lower than the US average of 6.5%^8^ but significantly higher than the 2.1% that it had been the year before.^9^

**Population Health Research Capsule**

What do we already know about this issue?

*COVID-19 has highlighted the importance of understanding the impact of social determinants of health on overall well-being.*

What was the research question?

*What were the effects of the COVID-19 pandemic on our patient population in the emergency department?*

What was the major finding of the study?

*The COVID-19 pandemic worsened food access (13.6% of patients) and financial troubles (24%), and non-Whites had 19-fold increased odds ratio than Whites for food access, and 15-fold for financial troubles, exacerbating disparities in social determinants of health.*

How does this improve population health?

*Understanding the challenges faced by our patient populations can serve as a guide on how to assist them more comprehensively.*

The social vulnerability index (SVI) is used by the CDC to help identify communities that may need support before, during, or after disasters. Counties are ranked from 0 to 1, with 1 being the highest vulnerability. Each theme is ranked, along with an overall SVI score assigned.^10^ The SVI scores show a low to moderate level of vulnerability at 0.2675 for our county (socioeconomic status 0.201; household composition and disability 0.006; minority status and language 0.7494; and housing/transportation 0.8593).^10^

### Participants

Participants included patients (or their guardians if the patient was <18 years of age) who were seen in the ED from October 2020–February 2021. It was administered within the ED to individuals selected by a convenience sample who were stable enough to give verbal consent and complete the form. Patients were not approached if they presented to the ED with unstable psychiatric or behavioral issues, altered mental status, or imprisonment status. Patients were excluded from the survey if they could not complete it in the following languages: Arabic; Chinese; English; French; Spanish; or Swahili.

### Outcomes

The primary outcomes of interest were food access and food concern before and during the COVID-19 pandemic. Food access was defined as food that was bought but didn’t last and the lack of money to buy more. Food concern was defined as individuals being worried that their food would run out before they got money to buy more. Secondary dependent variables included financial difficulty, anxiety, loss of interest, depression, stress, access to reliable transportation, living situation, safety, employment situation, substance use disorder, alcohol use disorder, use of tobacco and illegal drugs, disability, lack of exercise, and isolation before and during the COVID-19 pandemic. Each subject was asked to report whether these concerns got worse, better, or stayed the same during the COVID-19 pandemic compared to prior to the pandemic.

### Survey Instrument

The survey was adapted from the Accountable Health Communities Health-Related Social Needs Screening Tool (AHC HRSN) of the Centers for Medicare and Medicaid Services. The survey incorporated 13 specific domains to assess changes to participants’ social situations throughout the COVID-19 pandemic from January 2020–February 2021. We collected and managed study data from the survey using REDCap electronic data capture tools hosted at University of Iowa Hosptial and Clinics.^1^,^2^

### Measurements

After verbal consent was obtained, subjects completed the survey using an iPad. The survey response rate was measured based on the number of ED patients who were approached and the number who answered the survey during the study period. The AHC HRSN survey consisted of a total of 26 questions related to food, housing, transportation, access to healthcare, mental health, substance use, educational background, and physical activity using categorical responses. In addition to the participants’ answers to the AHC HRSN questions, we recorded the first two initials of their first name and the last two initials of their last name, along with their age, gender, race/ethnicity, and ZIP code.

A member of the research team opened the survey on the tablet and asked whether the patient preferred to complete the survey on their own or if they needed assistance. Patients who needed assistance included those who required mechanical assistance (pressing buttons or working a tablet, for example) or reading assistance. The survey was automatically uploaded to the database; no data entry was required of the research team.

### Study Size

The proposed study used hypothesis generation to determine the extent of food insecurity. Estimated food insecurity was 10% pre-pandemic and 20% post-pandemic.^12^ Given these numbers, a sample size of 199 was needed to have 80% power, 5% alpha, and 80% beta.

### Missing Data

We excluded missing observations from the analysis.

### Statistical Methods

Descriptive statistics are presented as frequencies and proportions. The primary outcome variables, which include food concern and food access, had three levels and were analyzed using multinomial logistic regression with “stayed the same” as the referent group. We adjusted the models for age, race, and gender. Age was categorized as ≤50 and >50 years, and race was grouped as White and non-White. Secondary outcomes were analyzed using binomial and multinomial logistic regression, as appropriate, adjusting for age, gender, and race.

Effect size was measured using unadjusted odds ratios (OR) and adjusted odds ratios (aOR) with 95% confidence intervals (CI). All tests were two-sided; statistically significant level was set at *P*-values less than 0.05. We performed statistical analyses using SAS version 9.4 (SAS Institute Inc, Cary, NC). Forest plot was displayed using Prism 9.0 (Graphpad Software, San Diego, CA).

## RESULTS

### Participants

We received 200 (66%) responses from the 305 patients who were approached for a survey. Of the 305 individuals approached, 105 did not agree to participate or were excluded. The predominant reasons for exclusion were lack of interest in completing a survey, followed by mandatory exclusion for prisoners; other patients who could not participate included those in isolation precautions, those who had completed the survey at a past ED visit, and those who declined due to high pain levels. Over two thirds of participants were White and over half were female. See table for participant characteristics.

### Main Results

Worsened food access was reported by 8.5% (17) of respondents, while 13.6 % (27) reported worsened food concern since the onset of the COVID-19 pandemic. Higher odds of worsened food access (OR 8.69, 95% CI 2.87- to 26.31), and worsened food concern (OR 10.39, 95% CI 3.79–28.49)] was reported by non-Whites when compared to Whites.

### Adjusted Odds Ratio for Food Access and Food Concern

The odds of worsened food access were higher among non-Whites (aOR 19.17, 95% CI 3.33–110.53) and females (aOR 9.77, CI 1.51–63.44). Also, non-Whites had greater odds of worsened food concern (aOR 15.31, CI 3.94–59.5) when compared to Whites.

### Secondary Analyses

Worsened financial difficulty was reported by 24% (48) of respondents. Higher odds of worsened financial difficulty was reported by patients aged ≤50 years or less compared to those >50 years (OR 2.46, 95% CI 1.11–5.48), Hispanics compared to non-Hispanics (OR 4.89, 95% CI 1.05–22.84] and non-Whites compared to Whites (OR 5.43, 95% CI 2.20–13.41). The odds of worsened financial difficulty were higher among females (aOR 2.87, 95% CI 1.08–7.65]), non-Whites (aOR 10.53, 95% CI 2.75–40.35]), and patients aged ≤50 years (aOR 2.48, 95% CI 1.00–6.14).

Worsened anxiety since the onset of the COVID-19 pandemic was reported in 30% (59) of individuals following the pandemic. No one reported worsened depression, and 20.8% (41) reported their depression had improved. Females had higher odds of worsened loss of interest or pleasure in doing things since the COVID-19 pandemic started (aOR 3.10, 95% CI 1.08–8.86) adjusted for age and race. The odds of having worsened difficulties concentrating and making decisions due to a physical, mental, or emotional condition in the COVID-19 period were higher for non-Whites (OR 4.00, 95% CI 1.07–14.90]. After adjusting for age and gender, non-Whites had more difficulties post COVID-19 pandemic with reliable transportation (aOR 25.37, 95% CI 3.99–161.20) adjusted for age and gender (Figure).

## DISCUSSION

The effect of the COVID-19 pandemic on SDoH highlights the importance of recognizing health and well-being beyond the limits of the healthcare system. It is important to acknowledge the vulnerabilities and strengths of communities. This allows for interventions that support health more fully. Overall, we found that the SDoH of our patients worsened during the pandemic. Participants were found to have increased concerns over food security, anxiety, and their financial situations. Moreover, non-Whites and females were often found to be disproportionately affected as compared to their White and male counterparts. Non-Whites were found to have higher odds of worsened food concern and access, financial difficulties, and transportation needs as compared to Whites. Females were also found to have higher odds of financial difficulty.

Food insecurity is a problem that affects numerous people living in the US. While the overall rate of food insecurity remained constant at 10.5% from 2019 to 2020, subgroup analysis showed that the rate of food insecurity for households with Black and non-Hispanic inhabitants rose from 19.1% to 21.7% per a report from the US Department of Agriculture.^13^ Our findings also found that non-Whites were disproportionately affected and had worsened food concern and access.

Not only did many participants have concerns related to food, but those surveyed also reported increased financial concerns. These findings may be reflective of the significant job loss that occurred following the pandemic.^14^,^15^ Our results indicate that 24% of participants experienced worsened financial difficulty since the start of the pandemic. A recent Canadian study also showed that about 20% of participants had concerns over meeting basic needs in the following six months of the pandemic.^16^

Furthermore, there were differences among vulnerable communities’ financial concerns. An April 2020 survey from the Pew Charitable Trust noted that 48% of Blacks, 44% of Hispanics, and 26% of White adults said they “cannot pay some bills or can only make partial payments on some of them this month.”^14^ In a typical month, 46% of Black, 28% of Hispanic, and 20% of Whites reported difficulty paying bills.^14^. We found that non-Whites had higher odds of financial difficulties (OR 5.43, CI 2.20–13.41). One cause may have been that vulnerable populations were more likely to suffer from wage loss as was seen in the Pew Charitable Trust survey, in which 61% of Hispanics, 44% of Blacks, and 38% of Whites noted wage losses in April 2020.^14^

Barriers to transportation have been noted to disproportionately affect vulnerable communities.^15^ We found that non-Whites had higher odds of worsened transportation difficulty during the pandemic. The concern with worsening access to transportation is that lack of transportation can lead to worse health outcomes due to missed outpatient visits and limited access to outpatient services, which in turn lead to higher ED use.^15^,^17^,^18^ From a review of 25 studies, it was found that 10–51% of patients noted lack of transportation as a barrier to healthcare.^19^

With regard to mental health-related questions, our study had mixed results. While there was a 30% increase in anxiety, depression seemed to improve by 20.8%. A recent study in an Australia ED found that presentations for anxiety and social and behavioral issues increased by 11.1% and 6.5%, respectively, but suicidal ideation and self-harm decreased by 26%.^20^ The authors of that study noted that while there was a decrease in the number of individuals presenting to the ED for suicidal thoughts, there was an increase in the use of mental health support hotlines.^20^ It is possible that our patients also chose other methods of receiving care during the pandemic regarding depressive symptoms.

It has been described throughout the pandemic that many people avoided visits to the ED due to concerns of encountering patients infected with COVID-19. An additional reason for the somewhat unexpected result with regard to mental health in our study could be due to the study methods we used. While we did not directly exclude patients with mental health issues, individuals who were not considered stabilized at the time of the sampling would not have been invited to participate. On further analysis, we also found that females had higher odds of worsened loss of interest or pleasure in doing things as compared to men. A study by Lindau et al evaluated the relationship in women between changes in health-related socioeconomic risks (HRSR) and mental health pre-pandemic and early in the pandemic phase. They found that women, regardless of previous HRSR, had worsened depression and anxiety early in the pandemic but that it was most prominent in those with high HRSRs.^21^

## LIMITATIONS

There are several limitations to consider. First, our study was largely exploratory. Second, prisoners and unstable patients were excluded. There may have been a selection bias from the research staff in approaching patients with mental health, behavioral, or intoxication chief complaints. It can be difficult to determine when these patients meet the criteria for being stable for participation; however, research staff were able to reach out to clinicians to help determine whether the patient was stable. Due to limited personal protective equipment, research staff were not always able to approach a patient suspected of having COVID-19.

Additionally, a non-response bias may have been present. Individuals who declined to participate may not have had similar characteristics to those who participated. For individuals who did participate, a recall bias may have been present. Participants were asked to think retrospectively about their social situations at the time the survey was completed. It should be noted, too, that our study may not be generalizable to other populations. Most of our patients are predominantly White and speak English. While we translated the survey into multiple other languages spoken by our patients, the survey was collected only from those who spoke English. Finally, our study took place in a single academic hospital in a largely rural Midwestern state and may not be representative of other geographical areas.

## CONCLUSION

The COVID-19 pandemic has worsened many of the social determinants of health found within the communities we serve. Moreover, women and non-Whites were found to have more financial and food concerns as compared to their counterparts. Understanding the challenges faced by our patient populations can serve as a guide on how to assist them more comprehensively. Interventions can then target resource-building and community partnerships that will help our patients get the support they need.

## Supplementary Information



## Figures and Tables

**Figure:**
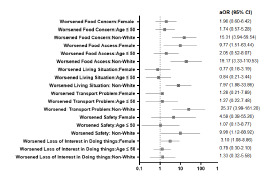
Odds Ratios of Additional Analysis *aOR*, adjusted odds ratio; *CI*, confidence interval.

**Table:** Characteristics of patients who completed a survey that assessed social risks.

Variables	Frequencies	Percent (%)
Ethnicity		
Hispanic or Latino	7	(4.0)
Not Hispanic or Latino	168	(94.9)
Unknown/not reported	2	(1.1)
Missing	23	
Race		
American Indian/Alaska Native	0	(0)
Asian	1	(0.6)
Native Hawaiian or other Pacific Islander	0	(0)
Black or African American	15	(8.6)
White	151	(86.3)
More than one	3	(1.7)
Unknown/not reported	5	(2.9)
Missing	25	
Age		
≤50	56	(48.3)
>50	60	(51.7)
Missing	84	
Gender		
Female		(55.6)
Male		(44.4)
Missing		

Footnote: The missing observations were not included in the percentages.
