# Supplementary material for: Impacts of the Pandemic on Social Determinants of Health in an Academic Emergency Department
Source: West J Emerg Med. 2022 Nov 1;23(6):811–6. doi: 10.5811/westjem.2022.8.56145 (PMC9683757; doi:10.5811/westjem.2022.8.56145)
Supplement: Supplementary file 1 [file wjem-23-811-s001.docx]

Supplemental Tables

Table 2: Primary Outcomes Variables

|  |  |  |  |  | Primary Outcome variables | | | | |  |  |  |
| --- | --- | --- | --- | --- | --- | --- | --- | --- | --- | --- | --- | --- |
|  |  |  |  | Food Access Since Onset of COVID-19 Pandemic | | | |  | Food Concern Since Onset of COVID-19 Pandemic | | | |
|  |  |  | Better | Worse | Same | Worse vs Same | Worse vs Same | Better | Worse | Same | Worse vs Same | Worse vs Same |
|  |  |  | n (%) | n (%) | n (%) | uOR (95%CI) | aOR (95%CI) | n (%) | n (%) | n (%) | uOR (95%CI) | aOR (95%CI) |
| **ALL** |  |  | 3(1.5) | 17(8.5) | 179(90.0) |  |  | 3(1.5) | 27(13.6) | 169(84.9) |  |  |
| Ethnicity |  |  | Col (%) | Col (%) | Col (%) | NA | NA | Col (%) | Col (%) | Col (%) | NA | NA |
| Hispanic or Latino | |  | 0(0) | 2(15.4) | 5(3.2) | 5.56(0.97 -32.02) | 1.33(0.07 -4.38) | 0(0) | 2(10.0) | 5(3.3) | 3.24(0.59 – 17.96) | 0.31(0.02 – 4.89) |
| Non-Hispanic or  Latino | | | 3(100.0) | 11(84.6) | 153(96.8) | 1.00 | 1.00 | 3(100.0) | 18(90.0) | 146(96.7) | 1.00 | 1.00 |
| Race |  |  |  |  |  |  |  |  |  |  |  |  |
| Caucasian | |  | 2(66.7) | 12(52.2) | 136(91.9) | 1.00 | 1.00* | 2(66.7) | 12(52.2) | 136(91.9) | 1.00 | 1.00* |
| Non-Caucasian | |  | 1(33.3) | 11(47.8) | 12(8.1) | 8.69(2.87 –26.31) | 19.17(3.33 – 110.53) | 1(33.3) | 11(47.8) | 12(8.1) | 10.39(3.79 – 28.49) | 15.310(3.937 – 59.537) |
| **Sex** |  |  |  |  |  |  |  |  |  |  |  |  |
| Male |  |  | 1(33.3) | 11(40.7) | 73(44.5) | 1.00 | 1.00* | 1(33.3) | 11(40.7) | 73(44.5) | 1.00 | 1.00* |
| Female |  |  | 2(66.7) | 16(59.3) | 91(55.5) | 2.70(0.85 – 8.62) | 9.77(1.51 – 63.44) | 2(66.7) | 16(59.3) | 91(55.5) | 1.17(0.51 – 2.67) | 1.96(0.60 – 6.42) |
| **Age** |  |  |  |  |  |  |  |  |  |  |  |  |
| ≤50 |  |  | 1(33.3) | 9(64.3) | 46(46.5) | 2.07(0.65 – 6.63) | 2.05(0.52 – 8.07) | 2(66.7) | 14(60.9) | 40(44.4) | 1.94(0.76 – 4.95) | 1.74(0.57 – 5.28) |
| 50 or older | |  | 2(66.7) | 5(35.7) | 53(53.5) | 1.00 | 1.00* | 1(33.3) | 9(39.1) | 50(55.6) | 1.00 | 1.00* |

^1^ Footnote:

All the variables in with the * were in the same model.

Ethnicity was controlled for race, sex and age in a separate mod

Table 3: Financial Difficulty

|  | Experienced Financial Difficulty | | | |
| --- | --- | --- | --- | --- |
|  | Yes | No |  |  |
|  | n (%) | n (%) | uOR (95%CI) | aOR (95%CI)^1^ |
| **ALL** | 48 (24.0) | 152 (76.0) | NA | NA |
| Ethnicity | Col (%) | Col (%) |  |  |
| Hispanic or Latino | 4(10.0) | 3(2.2) | 4.89(1.05 – 22.84) | 3.12(0.22 – 43.74) |
| Non-Hispanic or Latino | 36(90.0) | 132(97.8) | 1.00 | 1.00 |
| Race |  |  |  |  |
| Caucasian | 27(67.5) | 124(91.9) | 1.00 | 1.00* |
| Non-Caucasian | 13(32.5) | 11(8.2) | 5.43(2.20 – 13.41) | 10.53(2.75 – 40.35) |
| Sex |  |  |  |  |
| Male | 17(35.4) | 74(48.7) | 1.00 | 1.00* |
| Female | 31(64.6) | 78(51.3) | 1.88(0.94 – 3.17) | 2.87(1.08 – 7.65) |
| Age |  |  |  |  |
| <=50 | 23(48.9) | 32(47.8) | 2.46(1.11 – 5.48) | 2.48(1.00 – 6.14) |
| 50 or older | 24(51.1) | 35(52.2) | 1.00 | 1.00* |

^1^ Footnote:

All the variables in with the * were in the same model.

Ethnicity was controlled for race, sex and age in a separate model.

Table 4: Mental Health Effects

| Anxiety Since Onset of COVID-19 Pandemic | | | | | | | | Depression Worse Since Onset of COVID-19 Pandemic | | | | |
| --- | --- | --- | --- | --- | --- | --- | --- | --- | --- | --- | --- | --- |
|  |  |  | Better | Worse | Same | Worse vs Same | Worse vs Same | Yes | No |  | Yes vs No | Yes vs No |
|  |  |  | n (%) | n (%) | n (%) | uOR (95%CI) | aOR (95%CI) | n (%) | n (%) |  | uOR (95%CI) | aOR (95%CI) |
| ALL |  |  | 1(0.5) | 59(30.0) | 137(69.5) |  |  | 48(24.4) | 149(75.6) |  |  |  |
| Ethnicity |  |  | Col (%) | Col (%) | Col (%) | NA | NA | Col (%) | Col (%) |  | NA | NA |
| Hispanic or Latino | |  | 0(0) | 3(5.6) | 4(3.4) | 1.68(0.36 – 7.77) | 1.37(0.15 – 12.7) | 2(4.8) | 5(3.8) |  | 1.26(0.24 – 6.75) | 2.51(0.17 – 36.63) |
| Non-Hispanic or Latino | | | 1(100.0) | 51(94.4) | 114(96.6) | 1.00 | 1.00 | 40(95.2) | 126(96.2) |  | 1.00 | 1.00 |
| Race |  |  |  |  |  |  |  |  |  |  |  |  |
| Caucasian | |  | 0(0) | 10(18.9) | 14(11.8) | 1.00 | 1.00* | 5(11.9) | 19(14.5) |  | 1.00 | 1.00* |
| Non-Caucasian | |  | 1(100.0) | 43(81.1) | 105(88.2) | 1.74(0.72 – 4.23) | 1.76(0.57 – 5.45) | 37(88.1) | 112(85.5) |  | 1.26(0.44 – 3.60) | 0.50(0.08 – 3.13) |
| Sex |  |  |  |  |  |  |  |  |  |  |  |  |
| Male |  |  | 1(100.0) | 25(43.1) | 60(45.1) | 1.00 | 1.00* | 17(37.8) | 68(46.3) |  | 1.00 | 1.00* |
| Female |  |  | 0(0.0) | 33(56.9) | 73(54.9) | 1.09(0.58 – 2.02) | 1.28(0.57 – 2.99) | 28(62.2) | 79(53.7) |  | 1.41(0.72 – 2.81) | 2.82(1.08 – 7.38) |
| Age |  |  |  |  |  |  |  |  |  |  |  |  |
| ≤50 |  |  | 1(100.0) | 20(48.8) | 38(52.8) | 1.06(0.49 – 2.29) | 1.01(0.45 – 2.26) | 11(35.5) | 44(53.0) |  | 2.05(0.87 – 4.81) | 1.94(0.77 – 5.04) |
| 50 or older | |  | 0(0.0) | 21(51.2) | 34(47.2) | 1.00 | 1.00* | 20(64.5) | 39(47.0) |  | 1.00 | 1.00* |

^1^ Footnote:

All the variables in with the * were in the same model.

Ethnicity was controlled for race, sex and age in a separate model.

Table 5: Secondary Outcome Variables

| Secondary Outcome Variables | | | | | |
| --- | --- | --- | --- | --- | --- |
|  |  |  | Better | Worse | Same |
|  |  |  | n (%) | n (%) | n (%) |
| Since the COVID-19 Pandemic | | |  |  |  |
| **Living Situation (ALL)** | | | **5(2.5)** | **12(6.0)** | **182(91.5)** |
| Race |  |  | Col (%) | Col (%) | Col (%) |
| Caucasian | |  | 0(0) | 5(50.0) | 19(12.0) |
| Non-Caucasian | |  | 5(100.0) | 5(50.0) | 140(88.1) |
| Sex |  |  |  |  |  |
| Male |  |  | 2(40.0) | 7(58.3) | 76(42.9) |
| Female | |  | 3(60.0) | 5(41.7) | 101(57.1) |
| Age |  |  |  |  |  |
| <=50 |  |  | 2(40.0) | 7(58.3) | 47(47.5) |
| 50 or older | |  | 3(60.0) | 5(41.7) | 52(52.5) |
| **Transport Problem (ALL)** | |  | **3(1.5)** | **10(5.0)** | **186(93.5)** |
| Race |  |  |  |  |  |
| Caucasian | |  | 2(66.7) | 2(22.2) | 146(90.1) |
| Non-Caucasian | |  | 1(33.3) | 7(77.8) | 16(9.9) |
| Sex |  |  |  |  |  |
| Male |  |  | 1(33.3) | 5(50.0) | 79(43.7) |
| Female | |  | 2(66.7) | 5(50.0) | 102(56.4) |
| Age |  |  |  |  |  |
| <=50 |  |  | 3(100.0) | 5(62.5) | 48(45.7) |
| 50 or older | |  | 0(0) | 3(37.5) | 57(54.3) |
| **Safety (ALL)** |  |  | **1(0.5)** | **5(2.5)** | **191(97.0)** |
| Race |  |  |  |  |  |
| Caucasian | |  | 1(100.0) | 2(50.0) | 145(86.8) |
| Non-Caucasian | |  | 0(0) | 2(50.0) | 22(13.2) |
| Sex |  |  |  |  |  |
| Male |  |  | 1(100.0) | 1(20.0) | 82(44.1) |
| Female | |  | 0(0) | 4(80.0) | 104(55.9) |
| Age |  |  |  |  |  |
| <=50 |  |  | 0(0) | 3(60.0) | 51(47.2) |
| 50 or older | |  | 1(100) | 2(40.0) | 57(52.8) |
| **Loss of Interest Doing Things (ALL)** | | | **33(16.8)** | **3(1.5)** | **160(81.6)** |
| Race |  |  |  |  |  |
| Caucasian | |  | 25(86.2) | 1(50.0) | 122(86.5) |
| Non-Caucasian | |  | 4(13.8) | 1(50.0) | 19(13.5) |
| Sex |  |  |  |  |  |
| Male |  |  | 11(35.5) | 2(66.7) | 72(45.9) |
| Female | |  | 20(64.5) | 1(33.3) | 85(54.1) |
| Age |  |  |  |  |  |
| <=50 |  |  | 9(40.9) | 3(100.0) | 42(47.7) |
| 50 or older | |  | 13(59.1) | 0(0) | 46(52.3) |
